# Supplementary material for: MetaRibo-Seq measures translation in microbiomes
Source: Nat Commun. 2020 Jun 29;11:3268. doi: 10.1038/s41467-020-17081-z (PMC7324362; doi:10.1038/s41467-020-17081-z)
Supplement: Supplementary file 10 — Supplementary Data 7 [file 41467_2020_17081_MOESM10_ESM.zip › File2/Confidence_High_Taxonomy/220665_out.krona.html]

Javascript must be enabled to view this page.

members
magnitude
magnitudeUnassigned
count
unassigned
taxon
rank

220665\_out

351

351
superkingdom
2

1
phylum
1239

class
186801
1

1
order
186802

1
541000
family

1263
genus
1

2293181

SRS971276\_contig\_number\_11966
species
1

976
phylum
350

350
200643
class

171549
order
350

345
family
815

816
45

SRS013098\_contig\_number\_30371SRS013638\_contig\_number\_16876SRS015369\_contig\_number\_2819SRS015431\_contig\_number\_55760SRS016203\_contig\_number\_824SRS017103\_contig\_number\_33990SRS017701\_contig\_number\_9731SRS018427\_contig\_number\_12916SRS018836\_contig\_number\_16552SRS019286\_contig\_number\_5237SRS020271\_contig\_number\_contig-100\_129.130SRS020394\_contig\_number\_981SRS024331\_contig\_number\_17796SRS045739\_contig\_number\_3852SRS046502\_contig\_number\_12930SRS048981\_contig\_number\_2053SRS049959\_contig\_number\_41079SRS051610\_contig\_number\_10624SRS053398\_contig\_number\_22988SRS056259\_contig\_number\_2733SRS063489\_contig\_number\_3915SRS064557\_contig\_number\_3708SRS064757\_contig\_number\_22921SRS074586\_contig\_number\_2321SRS075773\_contig\_number\_17812SRS075821\_contig\_number\_3111SRS077194\_contig\_number\_13550SRS098655\_contig\_number\_12529SRS100021\_contig\_number\_2073SRS1041039\_contig\_number\_18939SRS1041129\_contig\_number\_14260SRS1041157\_contig\_number\_5179SRS104311\_contig\_number\_21372SRS144135\_contig\_number\_15563SRS144537\_contig\_number\_45182SRS144603\_contig\_number\_13293SRS146812\_contig\_number\_41745SRS147039\_contig\_number\_3650SRS147088\_contig\_number\_235SRS147557\_contig\_number\_32338SRS148091\_contig\_number\_12813SRS150029\_contig\_number\_5738SRS893230\_contig\_number\_7127SRS893300\_contig\_number\_11533SRS893378\_contig\_number\_3688
genus
345

1

SRS075021\_contig\_number\_563
species
357276

3

SRS077730\_contig\_number\_10221SRS098571\_contig\_number\_49752SRS893342\_contig\_number\_1514
species
817

1
204516

SRS097889\_contig\_number\_15305
species

329854

SRS024625\_contig\_number\_7893SRS104400\_contig\_number\_56933SRS1055043\_contig\_number\_11473SRS148319\_contig\_number\_11388
species
4

1
species

SRS143722\_contig\_number\_9585
1841855

29523
species

SRS064232\_contig\_number\_943
1

2

SRS015694\_contig\_number\_12191SRS143148\_contig\_number\_20854
species
2292928

species

SRS021948\_contig\_number\_15370
291644
1


SRS016095\_contig\_number\_2436
species
2292284
1

2
1262737

SRS046068\_contig\_number\_581SRS893380\_contig\_number\_471
species

1

SRS077024\_contig\_number\_3161
species
2292950


SRS011134\_contig\_number\_18632SRS011239\_contig\_number\_13613SRS011405\_contig\_number\_9753SRS012849\_contig\_number\_35660SRS012969\_contig\_number\_34858SRS013158\_contig\_number\_4271SRS013476\_contig\_number\_33533SRS013687\_contig\_number\_19976SRS013940\_contig\_number\_18054SRS013951\_contig\_number\_9599SRS014235\_contig\_number\_28327SRS014287\_contig\_number\_contig-100\_743.120822SRS014313\_contig\_number\_1432SRS014459\_contig\_number\_7619SRS014534\_contig\_number\_555SRS014683\_contig\_number\_6260SRS014855\_contig\_number\_5288SRS014923\_contig\_number\_27924SRS014948\_contig\_number\_22460SRS014979\_contig\_number\_36787SRS015065\_contig\_number\_28555SRS015190\_contig\_number\_10191SRS015217\_contig\_number\_28069SRS015578\_contig\_number\_31138SRS015663\_contig\_number\_16276SRS015794\_contig\_number\_8296SRS015854\_contig\_number\_13004SRS016056\_contig\_number\_482SRS016132\_contig\_number\_1043SRS016335\_contig\_number\_35533SRS016495\_contig\_number\_9767SRS016753\_contig\_number\_3540SRS016840\_contig\_number\_2193SRS016897\_contig\_number\_67SRS016954\_contig\_number\_19377SRS016989\_contig\_number\_4711SRS017247\_contig\_number\_9263SRS017307\_contig\_number\_15503SRS017521\_contig\_number\_30389SRS017745\_contig\_number\_692SRS017821\_contig\_number\_20062SRS018623\_contig\_number\_15880SRS018817\_contig\_number\_12573SRS018936\_contig\_number\_596SRS019030\_contig\_number\_7400SRS019381\_contig\_number\_1877SRS019397\_contig\_number\_801SRS019445\_contig\_number\_232SRS019496\_contig\_number\_11031SRS019601\_contig\_number\_20669SRS019808\_contig\_number\_18546SRS019910\_contig\_number\_9261SRS020328\_contig\_number\_2934SRS020508\_contig\_number\_contig-100\_173.21162SRS020622\_contig\_number\_5012SRS021153\_contig\_number\_1215SRS021484\_contig\_number\_4690SRS022071\_contig\_number\_22161SRS022524\_contig\_number\_9715SRS022609\_contig\_number\_27753SRS023526\_contig\_number\_34783SRS023583\_contig\_number\_438SRS023914\_contig\_number\_19407SRS023971\_contig\_number\_949SRS024009\_contig\_number\_2165SRS024075\_contig\_number\_11290SRS024132\_contig\_number\_24449SRS024435\_contig\_number\_9579SRS024663\_contig\_number\_9908SRS042284\_contig\_number\_462SRS042966\_contig\_number\_15398SRS043411\_contig\_number\_12288SRS044535\_contig\_number\_5533SRS045244\_contig\_number\_8210SRS045645\_contig\_number\_1901SRS045826\_contig\_number\_6515SRS046369\_contig\_number\_10426SRS047044\_contig\_number\_2312SRS047433\_contig\_number\_14352SRS048060\_contig\_number\_1186SRS048164\_contig\_number\_19789SRS048870\_contig\_number\_11696SRS049402\_contig\_number\_10083SRS049446\_contig\_number\_1359SRS049773\_contig\_number\_29973SRS050299\_contig\_number\_593SRS050422\_contig\_number\_182SRS050801\_contig\_number\_1086SRS050925\_contig\_number\_21166SRS050941\_contig\_number\_14181SRS050974\_contig\_number\_371SRS050998\_contig\_number\_12767SRS051031\_contig\_number\_25945SRS051882\_contig\_number\_9900SRS052027\_contig\_number\_17577SRS052576\_contig\_number\_contig-100\_931.932SRS053087\_contig\_number\_2491SRS053214\_contig\_number\_14246SRS053335\_contig\_number\_1748SRS054905\_contig\_number\_445SRS054956\_contig\_number\_16419SRS055017\_contig\_number\_1433SRS055982\_contig\_number\_1405SRS056537\_contig\_number\_40299SRS057480\_contig\_number\_9512SRS058723\_contig\_number\_2142SRS058770\_contig\_number\_23395SRS062427\_contig\_number\_8479SRS062654\_contig\_number\_14804SRS062701\_contig\_number\_144SRS063190\_contig\_number\_8479SRS063370\_contig\_number\_9248SRS064276\_contig\_number\_15462SRS064973\_contig\_number\_1658SRS065176\_contig\_number\_5294SRS074670\_contig\_number\_6507SRS075716\_contig\_number\_3900SRS075963\_contig\_number\_contig-100\_63.63SRS075984\_contig\_number\_2180SRS076804\_contig\_number\_250SRS076876\_contig\_number\_134SRS076976\_contig\_number\_2550SRS077086\_contig\_number\_1372SRS077231\_contig\_number\_24331SRS077294\_contig\_number\_702SRS077392\_contig\_number\_40060SRS077502\_contig\_number\_14344SRS077589\_contig\_number\_1567SRS077641\_contig\_number\_15811SRS077849\_contig\_number\_21677SRS078176\_contig\_number\_11094SRS078242\_contig\_number\_6271SRS078419\_contig\_number\_28285SRS098061\_contig\_number\_956SRS098073\_contig\_number\_3940SRS098644\_contig\_number\_26384SRS098717\_contig\_number\_6972SRS098881\_contig\_number\_15671SRS101268\_contig\_number\_48SRS101433\_contig\_number\_10163SRS1041031\_contig\_number\_27348SRS1041032\_contig\_number\_8584SRS1041033\_contig\_number\_16989SRS1041036\_contig\_number\_1702SRS1041037\_contig\_number\_8861SRS1041095\_contig\_number\_540SRS1041116\_contig\_number\_7428SRS1041118\_contig\_number\_1418SRS1041133\_contig\_number\_20143SRS1041137\_contig\_number\_14007SRS1041140\_contig\_number\_10619SRS1041141\_contig\_number\_5399SRS1041142\_contig\_number\_1044SRS1041143\_contig\_number\_7820SRS1041147\_contig\_number\_581SRS104693\_contig\_number\_2771SRS104975\_contig\_number\_7010SRS105153\_contig\_number\_12837SRS1054691\_contig\_number\_5480SRS1055022\_contig\_number\_10868SRS1055034\_contig\_number\_8477SRS1055038\_contig\_number\_5106SRS1055049\_contig\_number\_14540SRS1055056\_contig\_number\_1304SRS1055067\_contig\_number\_1501SRS1055069\_contig\_number\_9952SRS1055076\_contig\_number\_9364SRS1055095\_contig\_number\_96SRS140513\_contig\_number\_10505SRS142503\_contig\_number\_16841SRS142781\_contig\_number\_2350SRS142923\_contig\_number\_619SRS143085\_contig\_number\_1547SRS143181\_contig\_number\_627SRS143372\_contig\_number\_893SRS143466\_contig\_number\_7754SRS143523\_contig\_number\_10180SRS143598\_contig\_number\_1184SRS143991\_contig\_number\_17975SRS144183\_contig\_number\_23787SRS144297\_contig\_number\_6027SRS144506\_contig\_number\_40867SRS144714\_contig\_number\_27526SRS145308\_contig\_number\_2312SRS147271\_contig\_number\_8158SRS147377\_contig\_number\_1621SRS147653\_contig\_number\_1408SRS147766\_contig\_number\_498SRS147977\_contig\_number\_9518SRS148253\_contig\_number\_446SRS148424\_contig\_number\_32405SRS148721\_contig\_number\_22359SRS148784\_contig\_number\_13410SRS148817\_contig\_number\_10746SRS148970\_contig\_number\_929SRS149075\_contig\_number\_6699SRS149244\_contig\_number\_11612SRS149325\_contig\_number\_14549SRS149784\_contig\_number\_23518SRS892882\_contig\_number\_11096SRS893172\_contig\_number\_10851SRS893187\_contig\_number\_9600SRS893231\_contig\_number\_1284SRS893252\_contig\_number\_18196SRS893256\_contig\_number\_209SRS893279\_contig\_number\_436SRS893292\_contig\_number\_9109SRS893295\_contig\_number\_716SRS893341\_contig\_number\_10625SRS893358\_contig\_number\_2718SRS893366\_contig\_number\_18754SRS893369\_contig\_number\_8745SRS893373\_contig\_number\_27091SRS893382\_contig\_number\_946SRS893383\_contig\_number\_15498SRS971275\_contig\_number\_23494
species
820
216

291645

SRS076929\_contig\_number\_20827
species
1

1

SRS018541\_contig\_number\_2277
species
2086579


SRS011084\_contig\_number\_30598SRS015960\_contig\_number\_14721SRS063928\_contig\_number\_7SRS104636\_contig\_number\_14992SRS143417\_contig\_number\_25835SRS893253\_contig\_number\_356
species
28116
6


SRS077335\_contig\_number\_2072SRS143895\_contig\_number\_17592SRS147346\_contig\_number\_264
species
457393
3

3
species

SRS021219\_contig\_number\_9905SRS049712\_contig\_number\_14478SRS146764\_contig\_number\_30584
338188

species

SRS017433\_contig\_number\_9105SRS019161\_contig\_number\_9933SRS019968\_contig\_number\_9602SRS022137\_contig\_number\_17142SRS043768\_contig\_number\_13898SRS046717\_contig\_number\_308SRS049900\_contig\_number\_2982SRS076756\_contig\_number\_244SRS077454\_contig\_number\_839SRS077552\_contig\_number\_490SRS078665\_contig\_number\_1707SRS097920\_contig\_number\_1925SRS097958\_contig\_number\_1080SRS101376\_contig\_number\_12125SRS103987\_contig\_number\_5622SRS104327\_contig\_number\_282
46506
16

3
246787

SRS019267\_contig\_number\_8409SRS019685\_contig\_number\_18272SRS045713\_contig\_number\_19048
species

species

SRS065504\_contig\_number\_16668SRS142890\_contig\_number\_11153SRS143876\_contig\_number\_7466
47678
3

387090
species

SRS047014\_contig\_number\_1852
1

3
2292931
species

SRS014412\_contig\_number\_524SRS143342\_contig\_number\_11250SRS147614\_contig\_number\_1036

species

SRS015890\_contig\_number\_3889SRS017859\_contig\_number\_986SRS018984\_contig\_number\_17768SRS050752\_contig\_number\_25460SRS052697\_contig\_number\_32455SRS054059\_contig\_number\_570SRS1041091\_contig\_number\_2487SRS1041134\_contig\_number\_4784SRS1041138\_contig\_number\_9687SRS140492\_contig\_number\_28391SRS144362\_contig\_number\_11100SRS145497\_contig\_number\_602
93975
12

1

SRS064645\_contig\_number\_136
species
2292944

6
species

SRS043001\_contig\_number\_2327SRS065397\_contig\_number\_5524SRS077127\_contig\_number\_10630SRS1041112\_contig\_number\_5190SRS1041144\_contig\_number\_635SRS147425\_contig\_number\_502
818

species

SRS011302\_contig\_number\_3960SRS015782\_contig\_number\_376SRS050026\_contig\_number\_1083
310297
3

2
821

SRS019068\_contig\_number\_60352SRS142542\_contig\_number\_19697
species

2

SRS018133\_contig\_number\_9292SRS045004\_contig\_number\_15680
species
2292921

171552
family
1

577309
genus
1

1
1263095
species

SRS053649\_contig\_number\_4518

171550
family
1

1
239759
genus

28117

SRS1055099\_contig\_number\_77
species
1

family
1853231
1

283168
genus
1


SRS146813\_contig\_number\_742
species
626933
1

family
2005525
2

375288
genus
2


SRS015264\_contig\_number\_913
species
46503
1

1
species

SRS012273\_contig\_number\_32797
823
